# “Reverse engineering” research portfolio synergies and tradeoffs from domain expertise in minimum data contexts

**DOI:** 10.1371/journal.pone.0259734

**Published:** 2021-11-12

**Authors:** Benjamin Schiek

**Affiliations:** Alliance Bioversity-CIAT, Palmira, Valle del Cauca, Colombia; University of Almeria, SPAIN

## Abstract

In research portfolio planning contexts, an estimate of research policy and project synergies/tradeoffs (i.e. covariances) is essential to the optimal leveraging of institution resources. The data by which to make such estimates generally do not exist. Research institutions may often draw on domain expertise to fill this gap, but it is not clear how such ad hoc information can be quantified and fed into an optimal resource allocation workflow. Drawing on principal components analysis, I propose a method for “reverse engineering” synergies/tradeoffs from domain expertise at both the policy and project level. I discuss extensions to other problems and detail how the method can be fed into a research portfolio optimization workflow. I also briefly discuss the relevance of the proposed method in the context of the currently toxic relations between research communities and the donors that fund them.

## Introduction

Agricultural research for development (AR4D) institutions tend to give careful consideration to the formulation of their policies and strategic objectives, but very little, if any, consideration to the tradeoffs and synergies that may arise between policies. An institution may decide to simultaneously pursue, for example, food security and environmental sustainability as overarching strategic objectives, without considering the implicit tradeoffs between capital-intensive, high input agriculture, on the one hand, and pro-poor, agroecological agriculture, on the other. Such tradeoffs mean that progress towards one strategic objective can offset or even annul progress towards another. Conversely, there may be areas where the institution’s policies complement each other, generating synergies and enhancing impacts.

A parallel problem exists at the project level: careful consideration is often given to the potential impacts of individual research projects within the institution’s portfolio; but very little, if any, consideration is given to the tradeoffs and synergies that may arise between projects. AR4D institutions can usually draw on a wealth of domain expertise to shed light on these synergies and tradeoffs in a piecemeal fashion; but efforts to scale and quantify such ad hoc assessments—for example, through the Delphi Method or the Analytical Hierarchy Process [1]—are costly and time consuming. There are also inevitably gaps where domain experts are unable or hesitant to venture an estimate. For example: What is the synergy/tradeoff between a heat tolerant bean project and a digital agriculture project?

In this article, I propose a low cost, expedient method for “reverse engineering” synergies and tradeoffs at both the policy and project levels. Drawing on principal components analysis, I show how a project synergies/tradeoffs (a.k.a. correlation) matrix can be approximated based upon an expert survey of correlations between the institution’s projects and its policies. It turns out that the project level problem is mathematically dual to the policy level problem, such that the policy synergies/tradeoffs are also obtained in this process.

To build intuition and provide a proof of concept, I illustrate the reverse engineering method with a graphical example based on financial data. I then walk through an illustrative example of how the method applies in the AR4D context. I then discuss potential applications in plant breeding and research portfolio optimization. I close with a brief discussion of the proposed method’s relevance in the context of currently toxic relations between the research community and the donor community.

## “Reverse engineering” principal components analysis to deduce synergies and tradeoffs

### Signals from noise: Dimensional reduction of portfolios

In principal components analysis, a dataset *X* containing *τ* observations of *n* variables is distilled into a dataset *S* of just *m* < *n* transformed variables that capture the main tendencies and structure in the data. The data are always centered. If the variables in *X* follow diverse scalings and/or units of measurement (i.e. if apples are being compared to oranges), then *X* should also be scaled to unit variance. (See Abdi [2] for an introduction to principal components analysis.) The distilled matrix *S* is defined

$$\begin{array}{c} S=X\tilde{P} \end{array}$$ (1)

Where $\tilde{P}$ is a matrix containing the retained *m* leading eigenvectors of the full set of eigenvectors *P*, which is taken from the eigendecomposition of the data covariance matrix Σ_*XX*_ (Eq 2).

$$\begin{array}{c} \Sigma_{XX}=P\Gamma P^{\prime } \end{array}$$ (2)

Where Γ is the diagonal matrix of the eigenvalues of Σ_*XX*_.

From the definition (1), it follows that the columns of *S* are uncorrelated with each other, and that their variance is given by the retained *m* leading eigenvalues of the data covariance matrix.

$$\begin{array}{c} \begin{array}{cc} \Sigma_{SS} & =\frac{1}{n-1}S^{\prime }S\\ & =\frac{1}{n-1}{\tilde{P}}^{\prime }X^{\prime }X\tilde{P}\\ & ={\tilde{P}}^{\prime }\Sigma_{XX}\tilde{P}\\ & ={\tilde{P}}^{\prime }P\Gamma P^{\prime }\tilde{P}=\tilde{\Gamma } \end{array} \end{array}$$ (3)

Where, to be clear, $\tilde{\Gamma }$ is a diagonal matrix containing the retained *m* leading eigenvalues of the full eigenvalue matrix Γ, which is extracted from the eigendecomposition of the data covariance matrix.

The columns of the distilled matrix *S* are variously referred to as the principal components (PC), the PC scores, the factor scores, or the dimensions. When dealing with noisy time series, as in this article, they might just as well be referred to as the “signals”, in the sense that they are signals extracted from noise.

There then remains the question of what essential process these dimensions or signals *S* describe. This can be interpreted based on how correlated they are with the variables in *X*. These signal-variable correlations (*K*_*XS*_) are found by first finding their corresponding covariances (Σ_*XS*_).

$$\begin{array}{c} \begin{array}{cc} \Sigma_{XS} & =\frac{1}{n-1}X^{\prime }S\\ & =\frac{1}{n-1}X^{\prime }X\tilde{P}\\ & =\Sigma_{XX}\tilde{P}\\ & =P\Gamma P^{\prime }\tilde{P}\\ & =\tilde{P}\tilde{\Gamma } \end{array} \end{array}$$ (4)

Given the vector of standard deviations of the variables in *X* (call this ***σ***_*X*_), and the standard deviations of the signals *S* (call this ***σ***_*S*_), the signal-variable correlation matrix *K*_*XS*_ then follows as

$$\begin{array}{c} \begin{array}{cc} K_{XS} & =D{\left(\sigma_{X}\right)}^{-1}\Sigma_{XS}D{\left(\sigma_{S}\right)}^{-1}\\ & =D{\left(\sigma_{X}\right)}^{-1}\tilde{P}\tilde{\Gamma }D{\left(\sigma_{S}\right)}^{-1} \end{array} \end{array}$$ (5)

(Where the notation *D*(***σ***_*X*_) stands for a diagonal matrix with the vector ***σ***_*X*_ along the diagonal.) But the standard deviations of the signals ***σ***_*S*_ are just the square roots of the retained eigenvalues (recall Eq 3), so this reduces to

$$\begin{array}{c} \begin{array}{cc} K_{XS} & =D{\left(\sigma_{X}\right)}^{-1}\Sigma_{XS}{\tilde{\Gamma }}^{-\frac{1}{2}}\\ & =D{\left(\sigma_{X}\right)}^{-1}\tilde{P}\tilde{\Gamma }{\tilde{\Gamma }}^{-\frac{1}{2}}\\ & =D{\left(\sigma_{X}\right)}^{-1}\tilde{P}{\tilde{\Gamma }}^{\frac{1}{2}} \end{array} \end{array}$$ (6)

Moreover, if *X* is scaled to unit variance, then this further reduces to

$$\begin{array}{c} K_{XS}=\tilde{P}{\tilde{\Gamma }}^{\frac{1}{2}} \end{array}$$ (7)

The signal-variable correlations matrix *K*_*XS*_ is sometimes referred to as the “loadings” matrix, in the sense that it indicates how much each variable in *X* loads onto a given signal; or, vice versa, how much each signal loads onto a given variable. (The terminology varies in the literature. Some prefer to call *P* the loadings.) In keeping with this convention, and in order to reduce notational clutter, *K*_*XS*_ is henceforth relabeled *L*. That is to say,

$$\begin{array}{c} L=K_{XS}=D{\left(\sigma_{X}\right)}^{-1}\tilde{P}{\tilde{\Gamma }}^{\frac{1}{2}} \end{array}$$ (8)

Or, if *X* is scaled to unit variance,

$$\begin{array}{c} L=K_{XS}=\tilde{P}{\tilde{\Gamma }}^{\frac{1}{2}} \end{array}$$ (9)

Note that, in the latter case, the inner product of the loadings is equivalent to the signals covariance matrix Σ_*SS*_ derived in Eq 3.

$$\begin{array}{c} \begin{array}{cc} L^{\prime }L & ={\left(\tilde{P}{\tilde{\Gamma }}^{\frac{1}{2}}\right)}^{\prime }\tilde{P}{\tilde{\Gamma }}^{\frac{1}{2}}\\ & ={\tilde{\Gamma }}^{\frac{1}{2}}P^{\prime }\tilde{P}{\tilde{\Gamma }}^{\frac{1}{2}}\\ & =\tilde{\Gamma } \end{array} \end{array}$$ (10)

The inner product of the loadings is thus orthogonal when the data *X* are scaled to unit variance.

Note, moreover, that the data correlation matrix *K*_*XX*_ can be calculated as the outer product of the full set of loadings with itself.

$$\begin{array}{c} \begin{array}{cc} LL^{\prime } & =D{\left(\sigma_{X}\right)}^{-1}P\Gamma^{\frac{1}{2}}{\left(D{\left(\sigma_{X}\right)}^{-1}P\Gamma^{\frac{1}{2}}\right)}^{\prime }\\ & =D{\left(\sigma_{X}\right)}^{-1}P\Gamma^{\frac{1}{2}}\Gamma^{\frac{1}{2}}P^{\prime }D{\left(\sigma_{X}\right)}^{-1}\\ & =D{\left(\sigma_{X}\right)}^{-1}P\Gamma P^{\prime }D{\left(\sigma_{X}\right)}^{-1}\\ & =D{\left(\sigma_{X}\right)}^{-1}\Sigma_{XX}D{\left(\sigma_{X}\right)}^{-1}\\ & =K_{XX} \end{array} \end{array}$$ (11)

(Where *L*, in this instance, refers to the full set of loadings, $L=D{\left(\sigma_{X}\right)}^{-1}P\Gamma^{\frac{1}{2}}$, as opposed to the set of retained loadings, as defined in Eq 8.)

**Fig 1:**
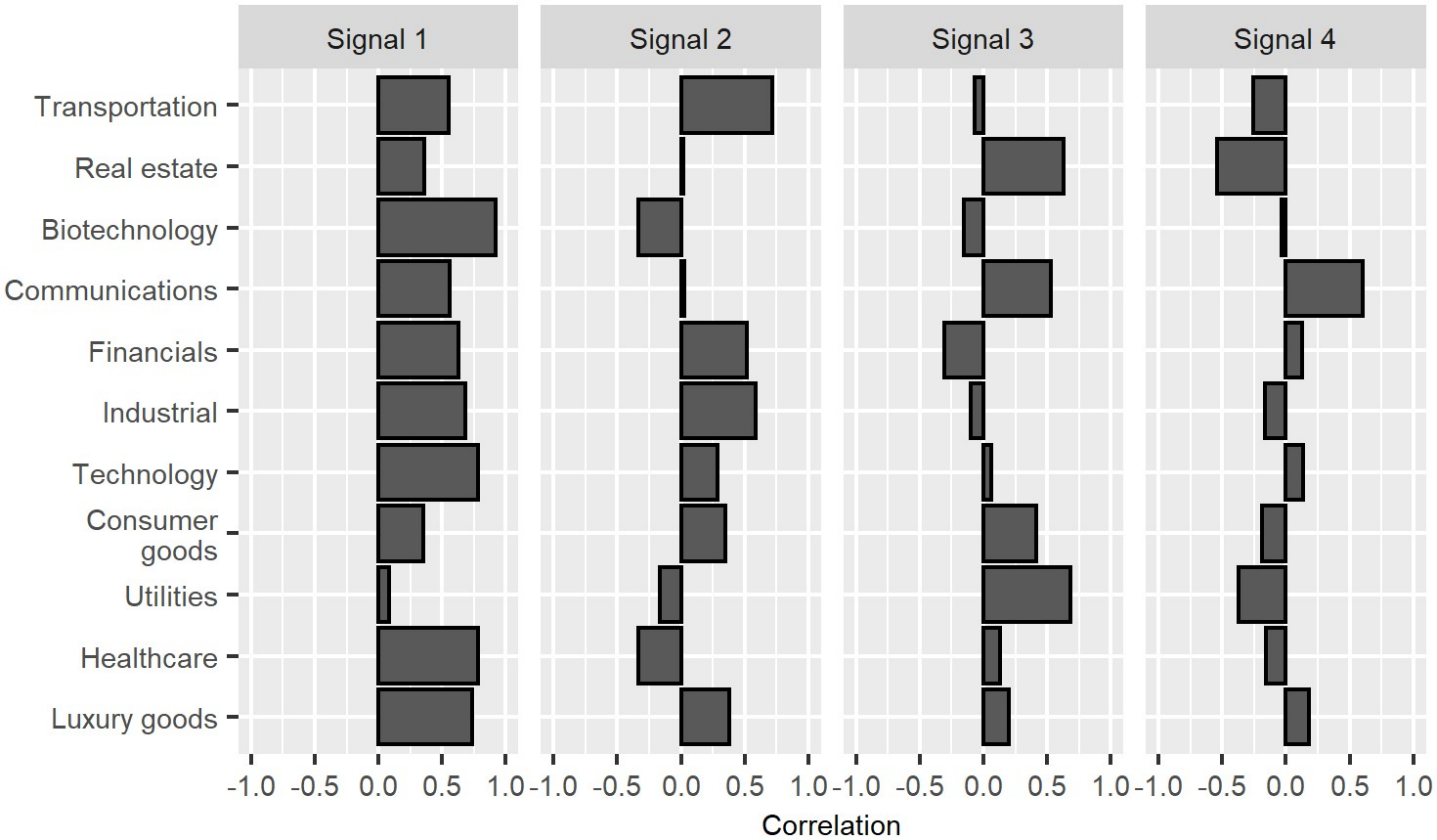
Correlation of variables (daily returns) with four leading signals extracted from the financial data.

An example of loadings is given in Fig 1. In this case, the variables are the daily returns of 11 financial securities covering the period 2019–01-29 to 2019–04-30, downloaded from yahoo finance using the R tidyquant package. The securities chosen for this example are exchange traded funds broadly representative of the U.S. economy. (See S1 Table for details.) The dataset is centered, but not scaled to unit variance. The signals are presented in descending order of their corresponding eigenvalues, with Signal 1 representing the principal component with the highest eigenvalue. The eigenvalue reflects the degree to which the signal describes the overall evolution of the data. Here, only the first four signals of the financial data set are shown. The question of how many signals should be extracted from the noise is addressed at the end of the section.

Concrete meaning can now be attributed to the otherwise abstract signals by examining the loadings—i.e. by examining how correlated the signals are with the variables. Signal 4, for example, appears to have something to do with price movements in Communications, and is negatively correlated with movements in the Real Estate sector. Signal 3, meanwhile, is positively correlated with Real Estate and Utilities, as well as Communications. Signal 3 might thus be loosely characterized as the “Housing and Urban Development” or “HUD” Signal, while Signal 4 might be called, rather convolutedly, the “Telecommunications Not Related to HUD” Signal. The interpretation of Signals 1 and 2 is still less straightforward, since they are both correlated with many variables.

### Applying an orthonormal rotation to clarify loadings

When the loadings are convoluted like this, it is useful to apply an orthnormal rotation to *L* in order to clarify the picture. That is to say, instead of examining *L*, one examines *L*_↺_.

$$\begin{array}{c} L_{↺}=LB \end{array}$$ (12)

Where *B* is the orthonormal rotation matrix, such that *B*′*B* = *I* and *BB*′ = *I*. It is important that *B* be orthonormal because the data correlation matrix *K*_*XX*_ is defined up to an orthonormal rotation of the full set of loadings. In other words, orthonormal rotations of the loadings leave the data correlation matrix unaltered. To see this, recall that the data correlation matrix can be calculated as the outer product of the full set of loadings with itself (Eq 11). And then note that this result is invariant under post multiplication of *L* by the orthonormal rotation matrix *B*.

$$\begin{array}{c} \begin{array}{cc} L_{↺}L_{↺}^{\prime } & =LB{\left(LB\right)}^{\prime }\\ & =LBB^{\prime }L^{\prime }\\ & =LL^{\prime }\\ & =K_{XX} \end{array} \end{array}$$ (13)

**Fig 2:**
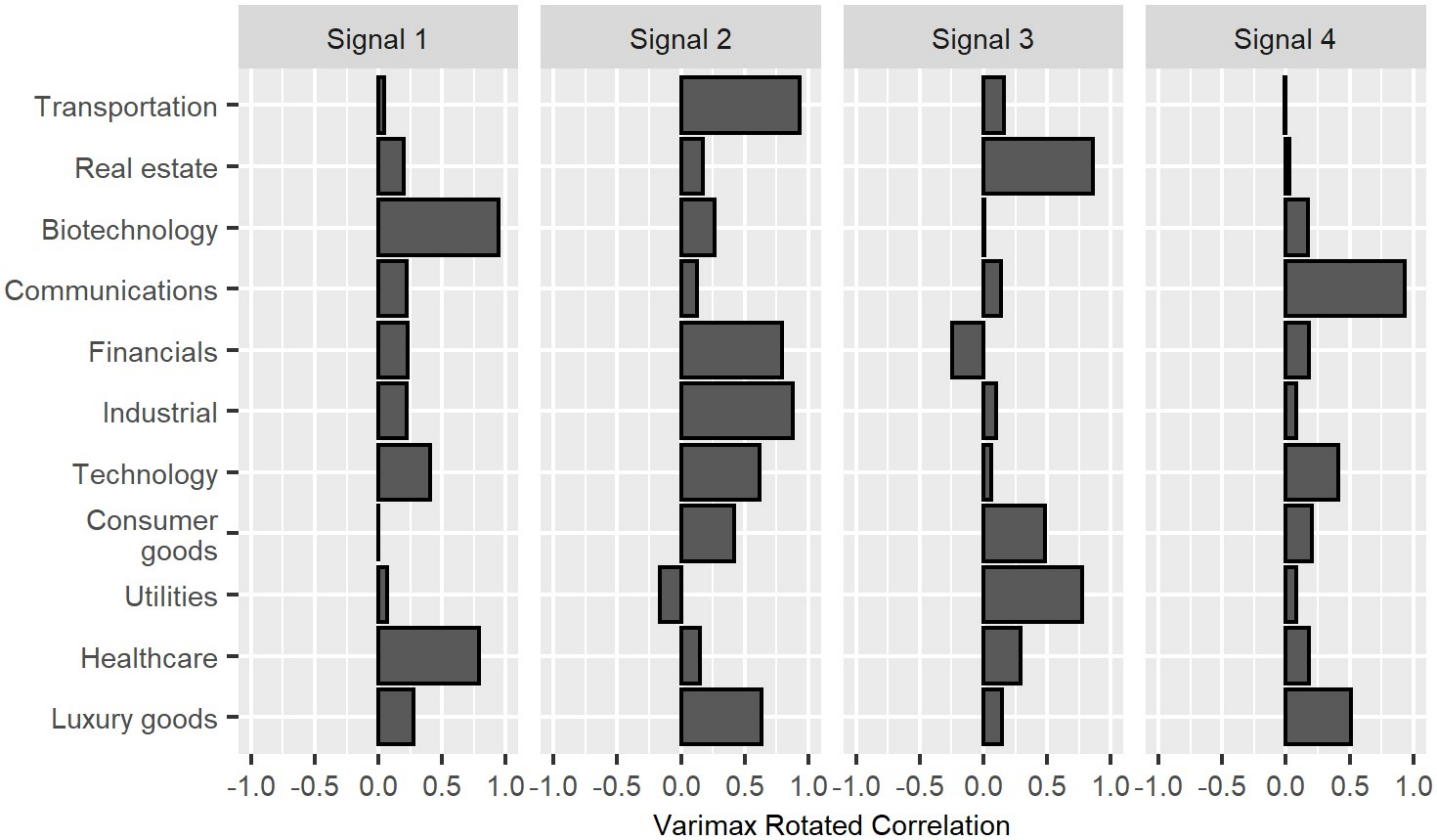
Varimax rotated correlation of variables with four leading signals.

In Fig 2 a special kind of orthonormal rotation, called a varimax rotation, is applied to *L*. Varimax rotations flesh out structure by maximizing sparseness in the rotated matrix. (For more details on varimax rotations, see Abdi [3].) After applying this rotation, Signal 1 is now clearly representative of Biotechnology and Healthcare, and so might be called the “Pharmaceutical” Signal. Signal 2 loadings are also now more distinctly pronounced, especially Financials, Industrial, and Transportation. Signal 2 might thus be called the “Financial and Physical Infrastructure” Signal. The rotation has also cleared up the overlap between Signals 3 and 4. Signal 4 is now more exclusively descriptive of price movements in the Communications sector and can thus be relabeled, more succinctly, the “Communications” Signal. Likewise, Signal 3 is now more exclusively descriptive of movements in Real Estate and Utilities, with some description of movements in the Consumer Goods sector.

Gopikrishnan, Rosenow, Plerou, and Stanley [4] pursued a similar line of inquiry when they looked at the components of the eigenvectors of a financial data correlation matrix. However, they did not explain that their findings are indicative of PC-asset correlations; nor did they apply an orthonormal rotation to clarify the interpretation.

**Fig 3:**
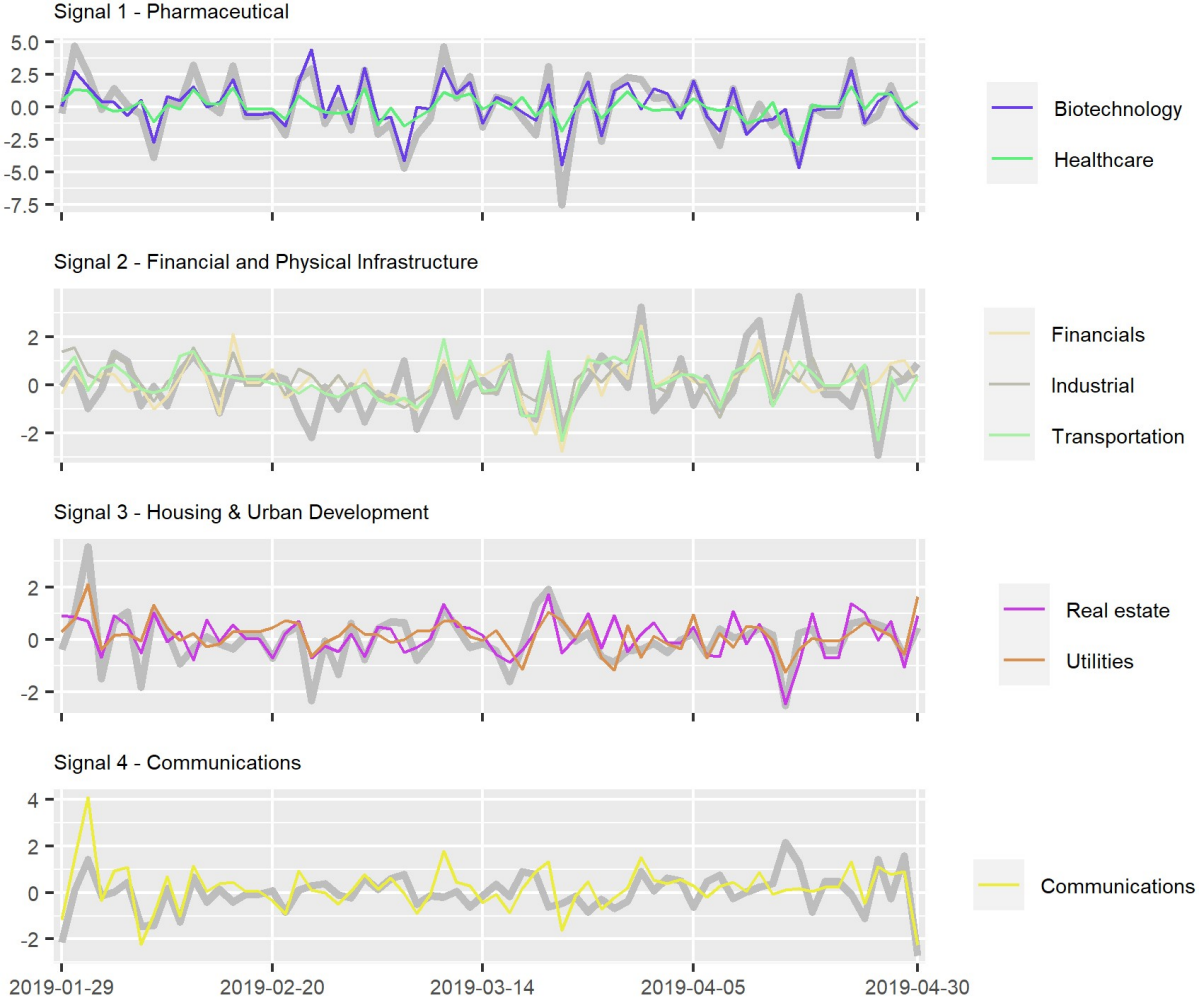
Signals (thick grey lines) plotted together with their most highly correlated variables.

Further visual confirmation of these interpretations of signal meaning is given by plotting the signals in the time domain together with their highest loading variables superimposed (Fig 3). Note how the highest loading variables hew closely to their respective signals.

If the data *X* is scaled to unit variance, then the orthonormal rotation matrix *B* and retained eigenvalues $\tilde{\Gamma }$ can be recovered from the eigendecomposition of the inner product $L_{↺}^{\prime }L_{↺}$.

$$\begin{array}{c} \begin{array}{cc} L_{↺}{}^{\prime }L_{↺} & =\left(\tilde{P}{\tilde{\Gamma }}^{\frac{1}{2}}B\right){}^{\prime }\tilde{P}{\tilde{\Gamma }}^{\frac{1}{2}}B\\ & =B{}^{\prime }{\tilde{\Gamma }}^{\frac{1}{2}}\tilde{P}{}^{\prime }\tilde{P}{\tilde{\Gamma }}^{\frac{1}{2}}B\\ & =B{}^{\prime }\tilde{\Gamma }B \end{array} \end{array}$$ (14)

Note the similarity to Eq 10. Whereas the inner product of the unrotated loadings *L*′*L* yields the signals covariance matrix Σ_*SS*_, the inner product of the orthonormally rotated loadings yields the covariance matrix of the orthonormally rotated signals (call this $\Sigma_{SS}^{↺}$). To see this, consider that, by definition,

$$\begin{array}{c} \begin{array}{cc} \Sigma_{SS}^{↺} & =\frac{1}{1-n}{\left(SB\right)}^{\prime }SB\\ & =\frac{1}{1-n}B^{\prime }S^{\prime }SB\\ & =\frac{1}{1-n}B^{\prime }{\left(X\tilde{P}\right)}^{\prime }X\tilde{P}B\\ & =\frac{1}{1-n}B^{\prime }{\tilde{P}}^{\prime }X^{\prime }X\tilde{P}B\\ & =B^{\prime }{\tilde{P}}^{\prime }\Sigma_{XX}\tilde{P}B\\ & =B^{\prime }{\tilde{P}}^{\prime }P\Gamma P^{\prime }\tilde{P}B\\ & =B^{\prime }\tilde{\Gamma }B \end{array} \end{array}$$ (15)

Therefore, $L_{↺}\prime L_{↺}=\Sigma_{SS}^{↺}$.

Moreover, having derived $\tilde{\Gamma }$ and *B* from the eigendecomposition of $L_{↺}^{\prime }L_{↺}$ via Eq 14, it is then possible to derive the retained leading eigenvectors of the data correlation matrix ($\tilde{P}$) as follows.

$$\begin{array}{c} \begin{array}{cc} L_{↺}B{}^{\prime }{\tilde{\Gamma }}^{-\frac{1}{2}} & =LBB{}^{\prime }{\tilde{\Gamma }}^{-\frac{1}{2}}\\ & =L{\tilde{\Gamma }}^{-\frac{1}{2}}\\ & =\tilde{P}{\tilde{\Gamma }}^{\frac{1}{2}}{\tilde{\Gamma }}^{-\frac{1}{2}}\\ & =\tilde{P} \end{array} \end{array}$$ (16)

When the data *X* are scaled to unit variance, then, the orthonormal rotation matrix *B*, retained eigenvalues $\tilde{\Gamma }$, and retained eigenvectors $\tilde{P}$ can be calculated from the rotated loadings *L*_↺_ alone, without any need for the original data *X*.

### How many signals to retain?

In practice, the number of signals that should be distilled from the original data set *X* depends upon how much of the variance in *X* the researcher wishes to capture or reflect in the signals, and how many signals are required to reach this subjectively determined threshold. The portion of the system’s evolution reflected in any given signal (call this *k*_*i*_) is defined as the signal’s variance divided by the sum of all signal variances. Recalling from Eq 3 that a signal’s variance is just the corresponding eigenvalue *γ*_*i*_ extracted from Σ_*XX*_, this is expressed

$$\begin{array}{c} k_{i}=\frac{\gamma_{i}}{\sum_{j=1}^{n}\gamma_{j}} \end{array}$$ (17)

The cumulative variance captured by a group of *m* < *n* signals is then

$$\begin{array}{c} c_{m}=\sum_{j=1}^{m}k_{j} \end{array}$$ (18)

Such that *c*_*n*_ = 1.

**Fig 4:**
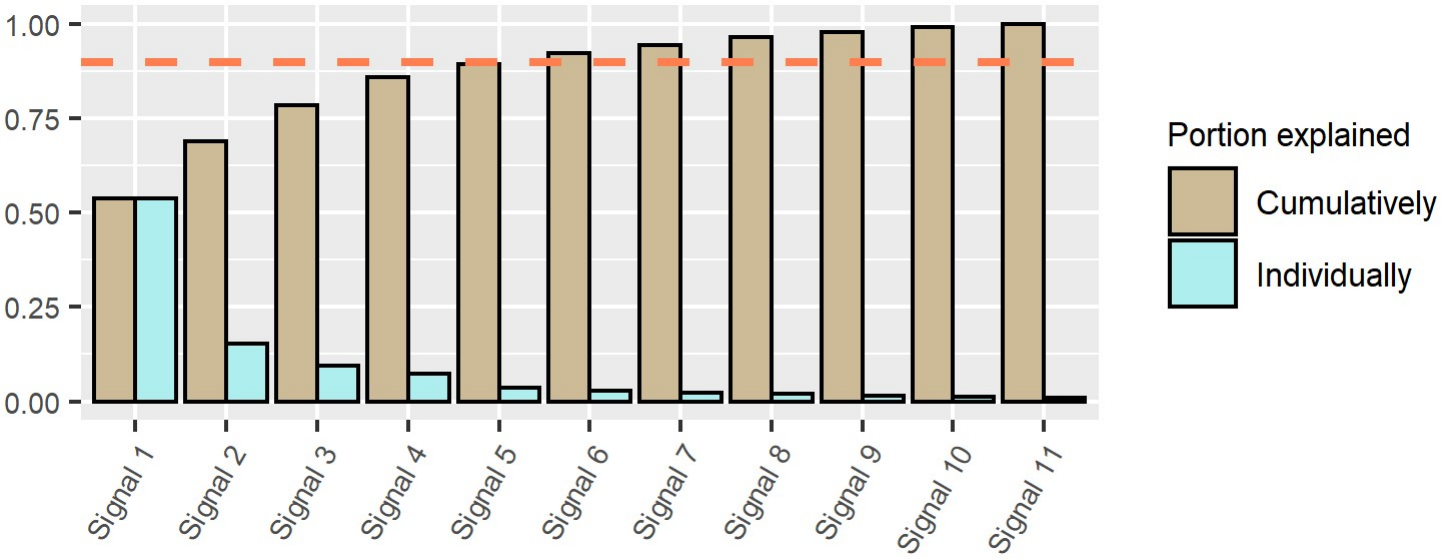
Plot of the individual and cumulative portions of variance explained by each signal in the financial dataset.

The individual and cumulative portions of variance explained by each signal in the financial dataset are plotted in Fig 4. Customarily, researchers like to retain signals such that at least 90% of the variance in the original data is explained. The horizontal dashed line in the plot marks this subjective threshold.

The plot shows that, for the financial data set, the leading 6 signals are sufficient to meet this criterion.

### Approximating the data correlation matrix from the retained loadings

Recall from Eq 11 that the data correlation matrix can be calculated as the outer product of the full set of loadings with itself. If *L* refers to the retained loadings only, the outer product of *L* with itself similarly yields an *approximate* data correlation matrix ${\tilde{K}}_{XX}$.

$$\begin{array}{c} \begin{array}{cc} LL^{\prime } & =D{\left(\sigma_{X}\right)}^{-1}\tilde{P}{\tilde{\Gamma }}^{\frac{1}{2}}{\left(\tilde{P}{\tilde{\Gamma }}^{\frac{1}{2}}\right)}^{\prime }D{\left(\sigma_{X}\right)}^{-1}\\ & =D{\left(\sigma_{X}\right)}^{-1}\tilde{P}{\tilde{\Gamma }}^{\frac{1}{2}}{\tilde{\Gamma }}^{\frac{1}{2}}{\tilde{P}}^{\prime }D{\left(\sigma_{X}\right)}^{-1}\\ & =D{\left(\sigma_{X}\right)}^{-1}\tilde{P}\tilde{\Gamma }{\tilde{P}}^{\prime }D{\left(\sigma_{X}\right)}^{-1}\\ & =D{\left(\sigma_{X}\right)}^{-1}\Sigma_{\tilde{X}X}D{\left(\sigma_{X}\right)}^{-1}\\ & ={\tilde{K}}_{XX} \end{array} \end{array}$$ (19)

Recall from Eq 13, moreover, that this operation is invariant under orthonormal rotation of the loadings, such that the outer product of the orthonormally rotated retained loadings $L_{↺}L_{↺}^{\prime }$ also gives the approximate correlation matrix ${\tilde{K}}_{XX}$.

**Fig 5:**
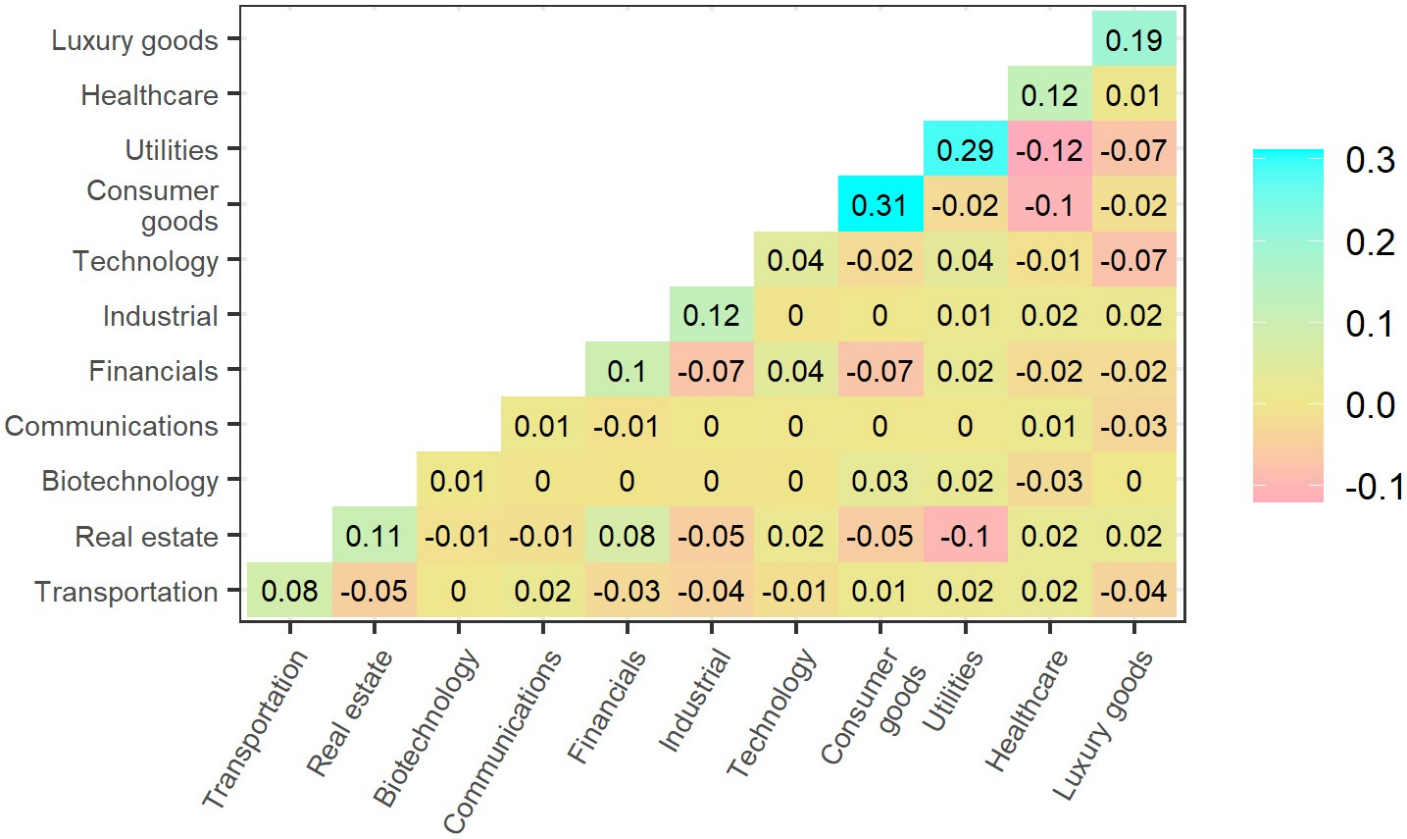
The financial data correlation matrix minus the approximate correlation matrix calculated as the outer product of the 6 leading loadings.

Even when just a handful of loadings are retained, ${\tilde{K}}_{XX}$ can approximate *K*_*XX*_ quite closely. The difference between the financial data correlation matrix and the approximate correlation matrix calculated from 6 retained loadings via Eq 19 is shown in Fig 5. Note that the difference is remarkably small for most entries.

The signals derived correlation matrix is approximate in the sense that it approximates the data correlation matrix; but it should not necessarily be considered inferior in terms of accuracy. To the extent that the original data are contaminated by noise, the approximate correlation matrix may prove more accurate with respect to the “true process” that generates the data.

### “Reverse engineering” the project correlation matrix from domain knowledge

The preceding sections demonstrate that it is possible to deduce quite a lot of information about a given centered and scaled dataset *X* from the rotated loadings *L*_↺_ alone, without any need to look at the original data. Specifically, Eqs 14 and 16 imply that it is possible to deduce from *L*_↺_ the retained eigenvalues and eigenvectors ($\tilde{\Gamma }$ and $\tilde{P}$), as well as the implicit orthonormal rotation matrix (*B*). Eq 19, meanwhile, demonstrates that an approximate data correlation matrix can be calculated as the outer product $L_{↺}L_{↺}^{\prime }$.

The question then naturally arises: Is it possible to deduce *L*_↺_ when *X* is not available?

In the AR4D context, if the columns of *X* are the unobserved (centered and scaled) expected returns to projects in a research institution’s research portfolio, and if the institution’s strategic objectives or policies may be considered analogous to a set of retained, leading, orthonormally rotated principal components (*SB*) describing 90% of the problem space that is of interest to the institution, then the correlations between institution policies and project expected returns may be interpreted as the orthonormally rotated loadings *L*_↺_ corresponding to *X*. An estimate of these policy-project correlations can then be elicited via a survey of domain experts at the institution and/or within its extended network of partners. And the outer product $L_{↺}L_{↺}^{\prime }$ may then be interpreted as the approximate project correlation matrix ${\tilde{K}}_{XX}$.

Importantly, the elicited policy-project correlations must be interpreted as *orthonormally rotated* loadings (*L*_↺_), as opposed to unrotated loadings (*L*), for two reasons: 1) The unrotated loadings are orthogonal (recall Eq 10), whereas the policy-project correlations crowdsourced from domain experts will generally not be orthogonal. 2) As seen in the financial example, orthonormally rotated loadings present a clearer picture of which variables are associated with which principal components than do the unrotated loadings. Generally speaking, AR4D institution policies are likewise formulated so as to be unique and distinct from each other. It therefore makes sense to interpret policies as orthonormally rotated principal components rather than unrotated principal components. Note that the implicit orthonormal rotation (*B*) could be a varimax rotation, as in the financial example, but does not necessarily have to be so. Correlation matrix invariance requires only that *B* be orthonormal (recall Eq 13).

If project risk (***σ***_*X*_) can be calculated beforehand during ex-ante impact assessment exercises, then it is straightforward to calculate an approximate project covariance matrix ${\tilde{\Sigma }}_{XX}$ from the elicited approximate correlation matrix ${\tilde{K}}_{XX}$ as follows.

$$\begin{array}{c} {\tilde{\Sigma }}_{XX}=D\left(\sigma_{X}\right){\tilde{K}}_{XX}D\left(\sigma_{X}\right) \end{array}$$ (20)

However, risk assessment is still not a standard part of ex-ante impact assessment models. (Alston and Norton acknowledged in 1995 that the treatment of risk in impact assessment models was “rudimentary and in need of further refinement” [5]. Unfortunately, this remains true today.) If ex-ante risk assessments are not available, then they can be elicited in the survey of domain experts. Project risk might be crowdsourced, for example, by asking survey participants to estimate the maximum, minimum, and most probable impact of each given project. With these three inputs, it is then straightforward to compute standard deviation on the basis of an assumed project impact probability density. (For example, the minimum and maximum could be interpreted as the bounds of the 95% confidence interval of a lognormal probability density, and the “most probable impact” could be interpreted as its mode. From this it is then straightforward to derive the standard deviation.)

### “Reverse engineering” the policy covariance matrix from domain knowledge

The interpretation of the orthonormally rotated signals *SB* as institution policies (or, more precisely, policy expected returns) implies that the orthonormally rotated signals covariance matrix $\Sigma_{SS}^{↺}$ may be interpreted as the policy covariance matrix. Recall from Eqs 14 and 15 that this matrix can be calculated as the inner product $L_{↺}\prime L_{↺}$.

With a crowdsourced *L*_↺_ in hand, then, it is possible to calculate both the approximate project correlation matrix and the the policy covariance matrix. Mathematically speaking, it can be said that the two calculations are dual to each other.

Note that the diagonal elements of the deduced policy covariance matrix are the policy variances consistent with the crowdsourced *L*_↺_. These may be interpreted as a measure of policy risk—i.e., the level of uncertainty surrounding expected policy returns—elicited from domain knowledge.

### When is it appropriate to “reverse engineer” correlation/covariance matrices?

The “reverse engineering” approach described above makes sense only in contexts where a relative lack of good data is compensated by a relative abundance of good domain knowledge. As a rule of thumb, the appropriateness of this approach may be assessed by meditating upon the conceptual ratio *ν*.

$$\nu =\frac{\text{confidence}\;\text{in}\;\text{domain}\;\text{knowledge}}{\text{confidence}\;\text{in}\;\text{data}}$$

As *ν* is higher, the reverse engineering approach makes more sense. As *ν* is lower, it becomes more appropriate to estimate the covariance matrix on the basis of data. For values of *ν* close to 1, a mixture of the two approaches might be considered. By this measure, the financial context is an inappropriate setting for the method proposed here, as financial data is abundant and financial experts are generally proven wrong on a daily basis (otherwise there would be a lot more billionaires in the world). On the other hand, AR4D contexts are an appropriate setting, as data regarding the value of research projects generally do not exist, but this is compensated by an abundance of scientific expertise.

## An illustrative example

In the example below, a hypothetical AR4D institution has the task of identifying synergies and tradeoffs in its project portfolio; and is also interested in quantifying any synergies and tradeoffs between its overarching policies. The institution’s projects are listed in Table 1. The unobserved time series of project returns may be thought of as the series of percentage changes in the project’s net present value per appropriate time step (quarterly, yearly, etc.). The projects are loosely grouped into four categories to facilitate interpretation of the subsequent graphics, but there is no strict rule followed, and clearly some overlap, in the grouping.

**Table 1:** Hypothetical list of AR4D projects.

Project	Group
Mega Maize	High Value Yield Enhancement
Hyper Rice	High Value Yield Enhancement
Ultra Cow	High Value Yield Enhancement
Cassava for Bio-ethanol	Smallholder Resilience
Triple Purpose Sweet Potato	Smallholder Resilience
Dairy Cooperative	Smallholder Resilience
Multi-stakeholder Platforms	Smallholder Resilience
Heat Tolerant Beans	Climate Smart Agriculture
Coffee Agroforestry	Climate Smart Agriculture
Digital Agriculture	Climate Smart Agriculture
Low Emission Silvopastoral	Climate Smart Agriculture

The institution’s strategic objectives or policies in this example are “Economic Growth”, “Income Equality”, “Environmental Sustainability”, and “Nutritional Security”, which roughly correspond to UN Sustainable Development Goals 8, 1, 13, and 3, respectively. Policy-project correlations are elicited via a survey of domain experts and/or stakeholders. Literature may also be consulted.

It should be clearly explained to survey participants that a positive policy-project correlation means the project contributes toward the policy (i.e. is a synergy), while a negative correlation means the project works against it (i.e. is a tradeoff); and a correlation of zero means that the project has no influence upon the given policy one way or the other. The language used in this survey should be familiar to participants. In most AR4D resource allocation settings, what I characterized in the financial example above as “signals” should probably be referred to as “policies”, “strategic objectives”, “criteria”, or simply “goals”.

Survey participants should also be encouraged to keep in mind that no AR4D project can “be all things to all people”. A new yield enhancing variety of a high value crop, for example, might contribute towards increased trade competitiveness and GDP growth, but at the cost of increased deforestation and use of chemical inputs that degrade the environment. Conversely, a climate smart or pro-poor AR4D proposal might increase long term environmental and socio-economic sustainability at the cost of reduced short-medium term growth and competitiveness. These tradeoffs require careful consideration. Participants might also be encouraged to beware of any received wisdom regarding tradeoffs and synergies. For example, it is customary in AR4D communities to assume that economic growth and economic equality are mutually exclusive goals [5], whereas recent empirical research suggests a much more nuanced and synergistic relation [6].

**Fig 6:**
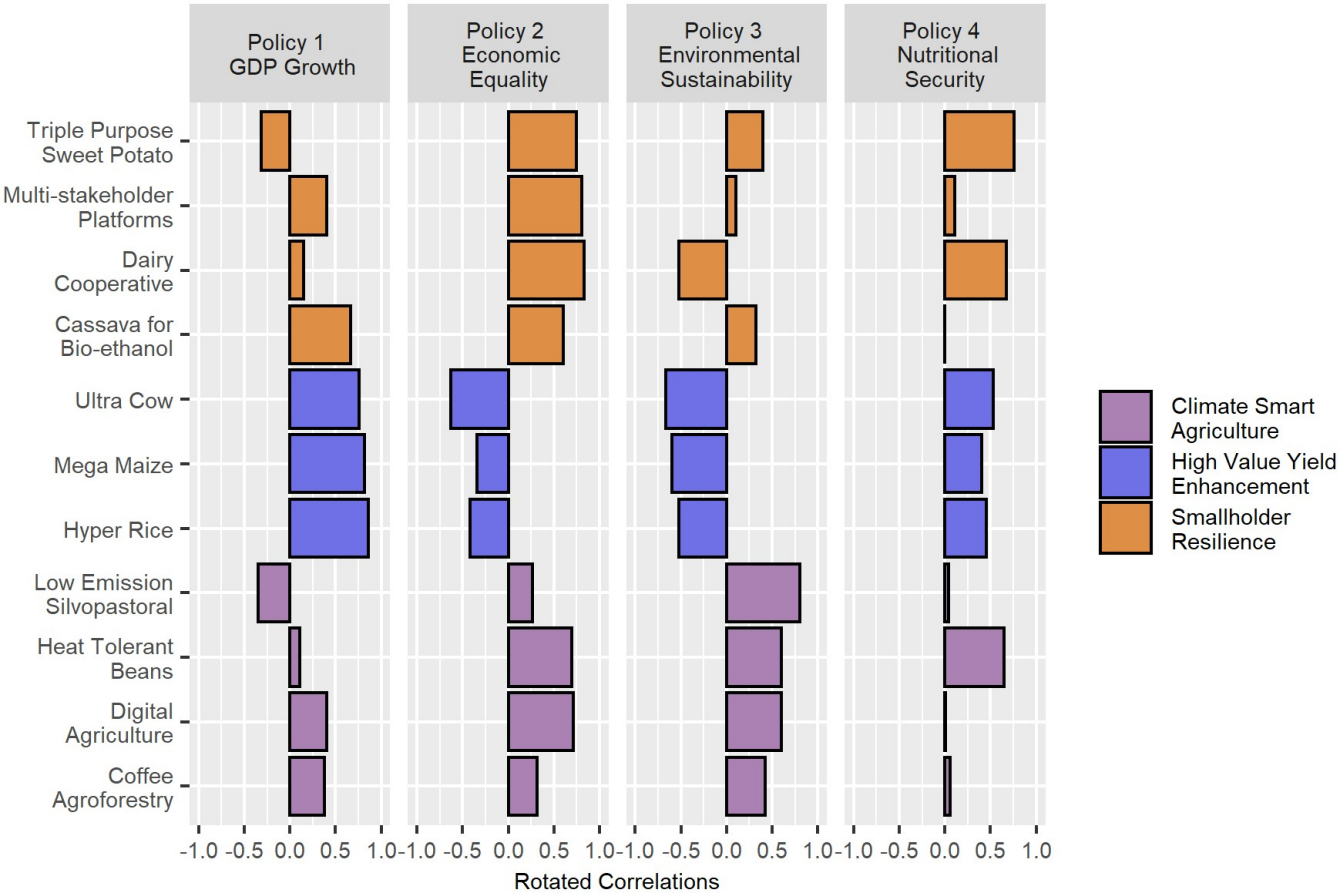
Hypothetical results of a survey eliciting policy-project correlations from experts and stakeholders.

The results of the survey are summarized in Fig 6.

The survey exercise concludes. The resulting crowdsourced policy-project correlations are then interpreted as the orthonormally rotated loadings *L*_↺_. An approximate project correlation matrix ${\tilde{K}}_{XX}$ is then reverse engineered from this domain knowledge as the outer product $L_{↺}L_{↺}^{\prime }$ (see Eq 19 for details). Since this is a correlation matrix, the diagonal elements must equal 1. However, as seen in the financial example (Fig 5), the diagonal elements of correlation matrices approximated from a retained set of loadings (whether crowd- or data-sourced) can diverge somewhat from 1. In order to correct for this divergence, the approximate project correlation matrix deduced from the crowdsourced loadings is divided through by its diagonal elements.

**Fig 7:**
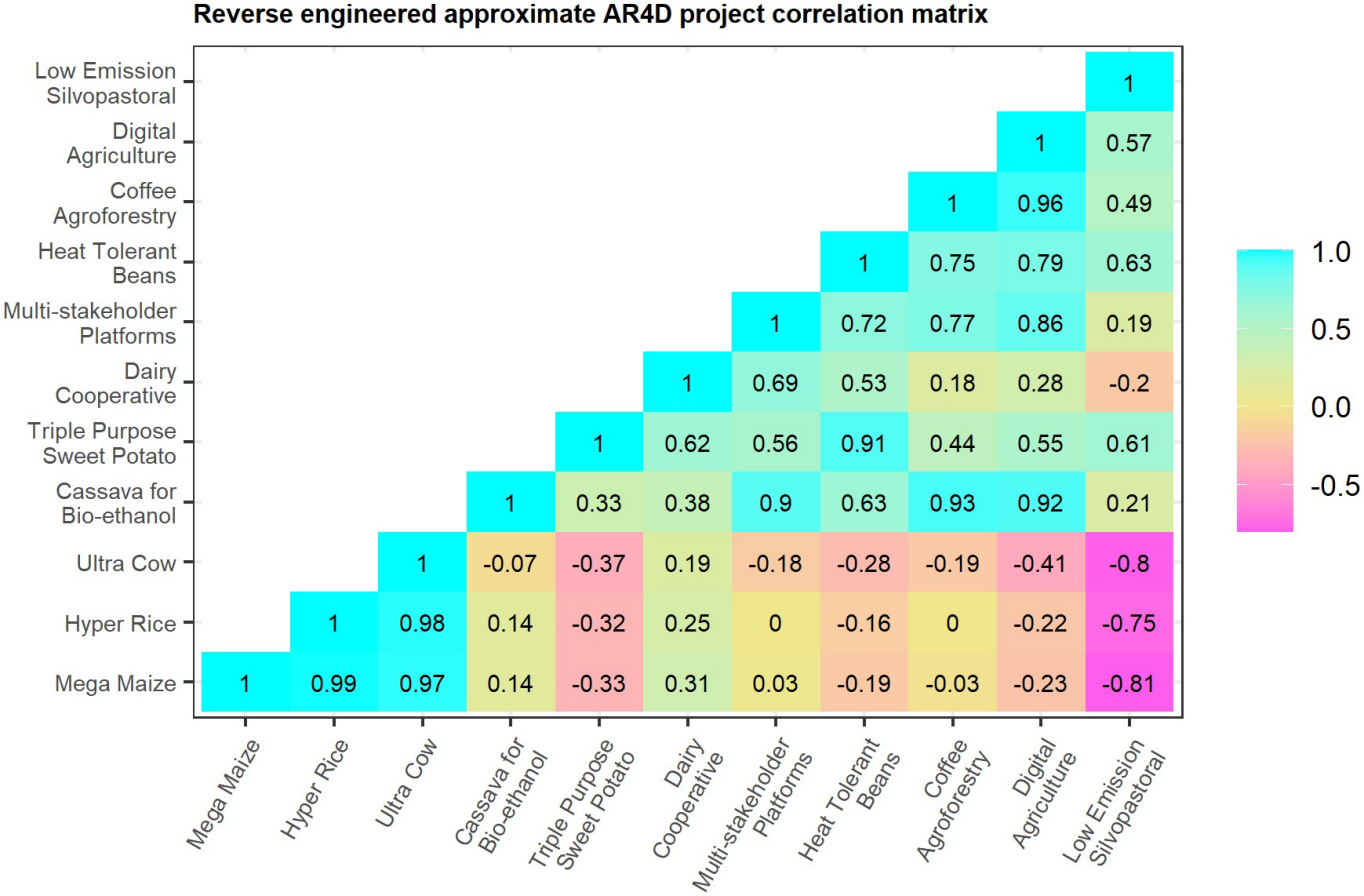
Approximate AR4D project correlation matrix calculated from the crowdsourced policy-project correlations.

The approximate project correlation matrix, displayed in Fig 7, can then be used to orient stakeholder discussions regarding tradeoffs and synergies between projects. Some of the matrix elements may serve to confirm expectations, while other elements may come as a surprise, or serve to fill in a gap where experts are hesitant to venture an estimate. It probably comes as no surprise to the hypothetical survey participants, for example, that the high yielding, high value AR4D projects (Hyper Rice, Mega Maize, and Ultra Cow) are strongly correlated with each other, or that they are negatively correlated with some of the climate smart projects (the Low Emission Silvopastoral proposal, in particular). On the other hand, few experts would be willing to venture an assessment of the synergy or tradeoff between the Cassava for Bio-ethanol and Coffee Agroforestry projects. The deduced covariance matrix effectively fills in such gaps with values that maximize consistency with the domain knowledge captured by the survey.

**Fig 8:**
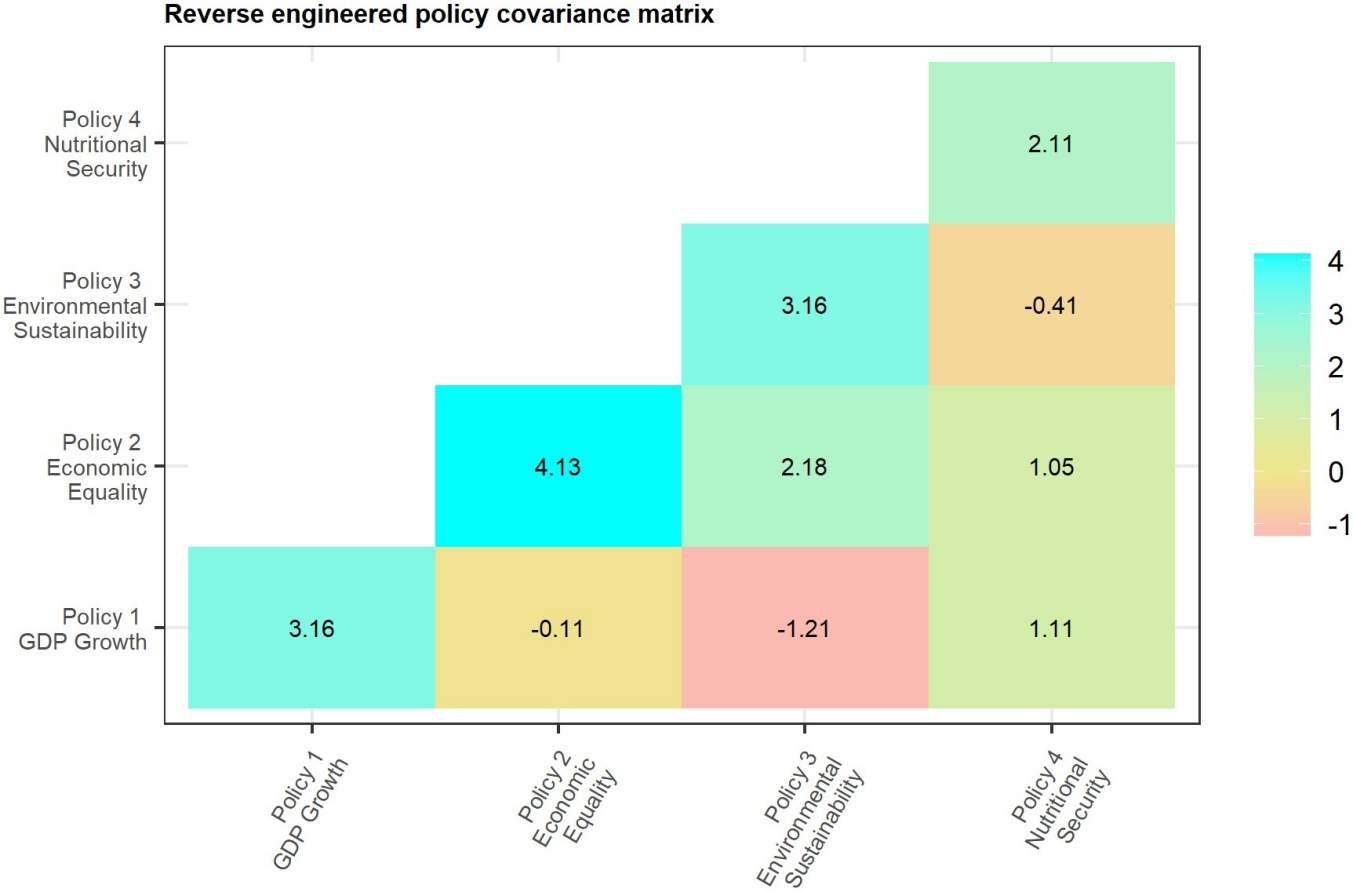
Policy covariance matrix calculated from the crowdsourced policy-project correlations.

The policy covariance matrix $\Sigma_{SS}^{↺}$, meanwhile, is reverse engineered from domain knowledge as the inner product $L_{↺}^{\prime }L_{↺}$ (see Eqs 14 and 15 for details). This matrix, displayed in Fig 8, can likewise be useful in orienting discussion regarding tradeoffs and synergies between policies. For example, there is often debate over whether or not, and by how much, Economic (GDP) Growth and Economic Equality policies might offset each other. The crowdsourced policy covariance matrix in this example indicates only a small tradeoff (negative covariance) of −0.11 between these two policy aims. The research institution might also be concerned about the compatibility of its Economic Growth and Environmental Sustainability policies. The matrix suggests these concerns are well-founded, indicating a considerable tradeoff of −1.21 between the two policies. Or, the institution may simply wish to identify its largest policy synergy. The crowdsourced matrix indicates that the institution’s largest synergy exists between its Environmental Sustainability and Economic Equality policies. This can then inform strategic discussions regarding the role, scale, design, and linkages of, say, pro-poor, agroecological initiatives, so as to best capitalize upon this synergy.

In this way, the off diagonal elements of the reverse engineered policy covariance matrix equip the research institution with a guide by which to capitalize on synergies while mitigating tradeoffs. Note that the diagonal elements of the matrix also provide important information. As noted earlier, these are the implicit policy variances, and may thus be interpreted as a measure of policy risk. The matrix in this example indicates that Economic Equality is the institution’s riskiest policy, while Nutritional Security is the least risky policy. Such information can aid the institution in identifying areas where risk mitigation measures may be necessary, and/or where contingency plans should be prepared in case projects do not unfold as expected.

## Discussion

The reverse engineering approach proposed above offers a perspective on otherwise unquantifiable project and policy synergies and tradeoffs. The accuracy of this perspective depends on 1) how completely the policies capture or describe the evolution of projects within the problem space (in the precise sense of Eq 18); and 2) the accuracy of the domain knowledge whence policy-project correlations are deduced. Fig 5, from the financial example, demonstrates that the crowdsourced project correlation matrix will closely match the (unobservable) data derived project correlation matrix insofar as these two conditions are fulfilled. It is thus important to apply this method in contexts where there is a high degree of confidence in domain knowledge compensating a general lack of good data (i.e. a high *ν* ratio). The method is open to criticism insofar as the domain knowledge is skewed by institutional inertia, politicized thinking, and other sources of subjective bias. However, the alternative method of data based estimation does not necessarily have a comparative advantage in this respect, as it is likewise subject to a host of different, but no less problematic, sources of bias and error.

Regardless of accuracy, the proposed method may have value as a consensus building tool regarding synergies and tradeoffs about which expert opinions differ or are lacking altogether. The method fills in such gaps with the values that effectively maximize consistency with the expert knowledge captured by the survey. In this process, the method may confront experts and stakeholders with potentially surprising logical implications of what they (think they) know about the problem space, and about the evolution of projects and policies through that space, thereby stimulating policy debate and dialogue.

The proposed method also provides a way to deduce the implicit retained eigenvalues and eigenvectors corresponding to the unobserved data, as well as the implicit orthonormal rotation matrix (recall Eqs 14 and 16 for details). While this information is not needed in the deduction of the approximate project correlation matrix, nor in the deduction of the policy covariance matrix, it may nonetheless prove useful for other research portfolio planning purposes not contemplated in this article.

### Potential application in plant breeding decision pipelines

Applications of the method presented here are not limited to the assessment of policies and projects. Another potential area of application within the AR4D arena, for example, is in the assessment of plant trait and variety correlations.

Plant breeders are typically tasked with the development of new varieties featuring a particular new trait—say, for example, resistance to a particular pest or disease—as well as numerous other traits such as fast maturation time, a particular taste, color, shape, nutritional content, and so on. In this process, a map of synergies and tradeoffs between traits and between varieties may be useful in guiding selection decisions.

**Fig 9:**
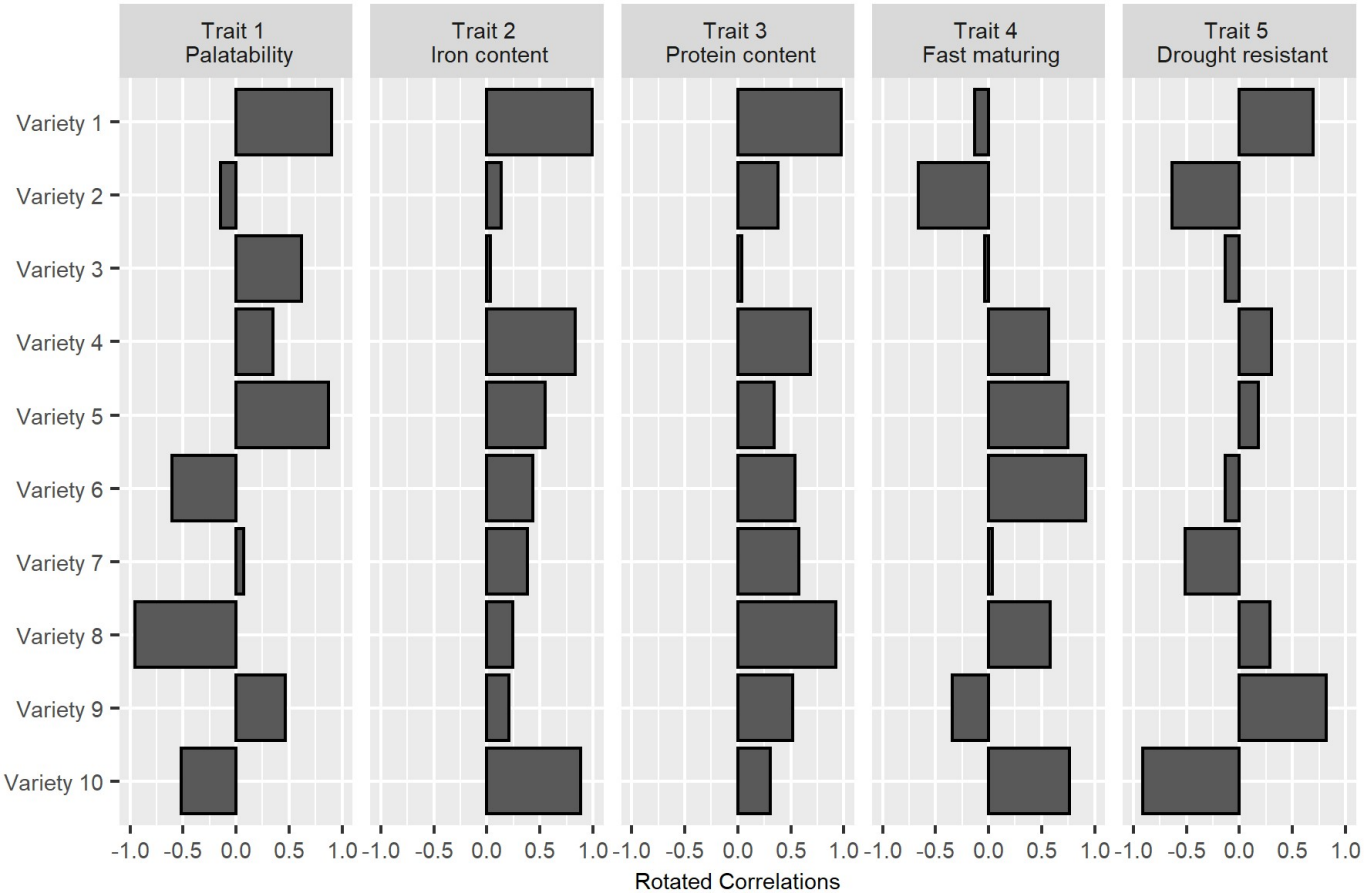
Hypothetical example of crowdsourced, orthonormally rotated trait-variety correlations.

**Fig 10:**
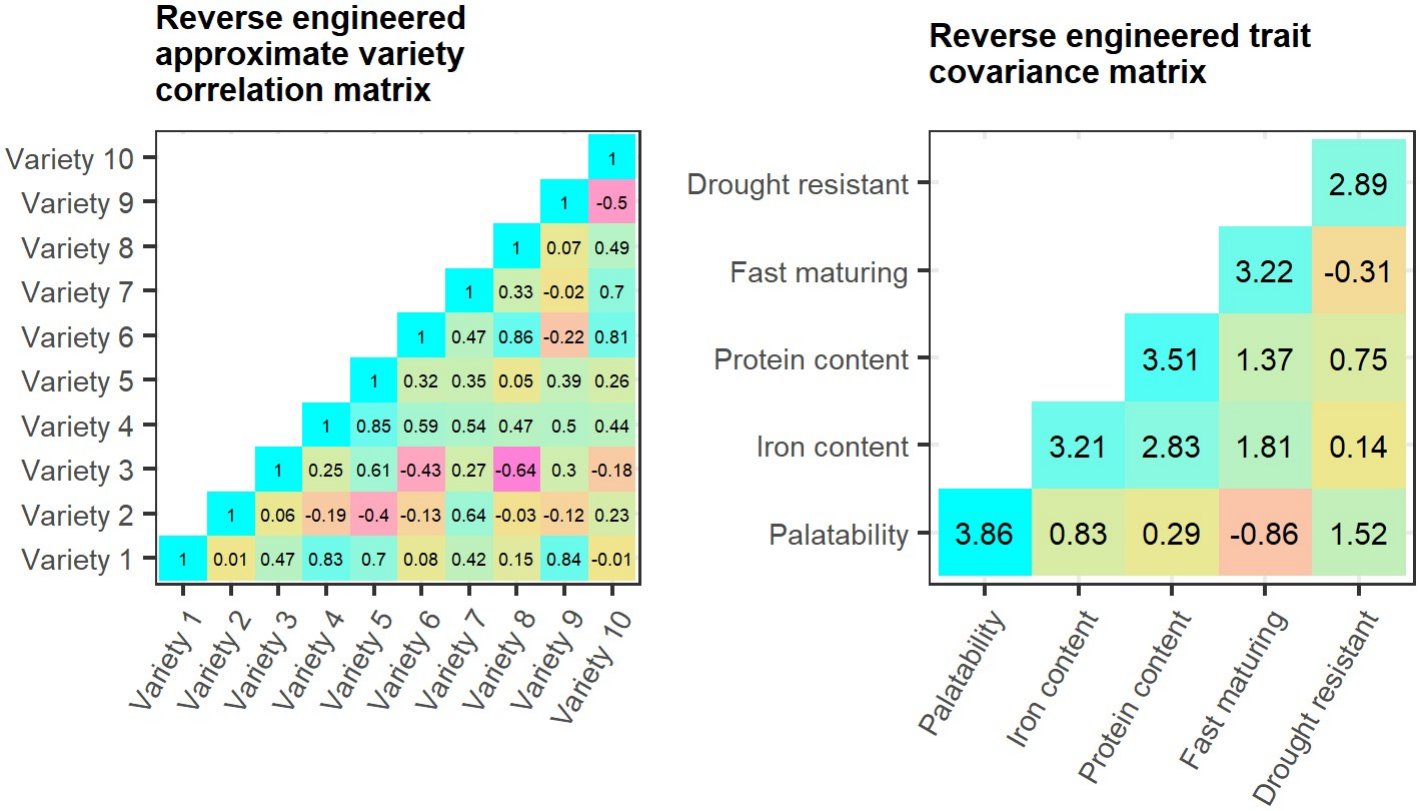
(Left) Approximate variety correlation matrix and (Right) trait covariance matrix reverse engineered from the hypothetical crowdsourced trait-variety correlations.

In this setting, varieties play the role that projects do in the previous example, while traits are analogous to the set of orthonormally rotated, retained principal components describing 90% of the problem space. Correlations between varieties and traits are elicited through a survey of breeding experts. A hypothetical example of such a crowdsourcing exercise for beans is given in Fig 9. The approximate variety correlation matrix and trait covariance matrix are then reverse engineered from this information in Fig 10.

### Potential application in research portfolio risk minimization

Some may be tempted to use the reverse engineered project covariance matrix in a portfolio risk minimization problem, so as to solve for the institution’s risk minimizing resource allocation across projects. For example, if the expected logged project portfolio utility is defined

$$\begin{array}{c} E\left[\text{ln}\left(U\right)\right]=E\left[X\right]{}^{\prime }\text{ln}\left(w\right) \end{array}$$ (21)

Where *E*[*X*] is the vector of column means of the unobserved expected project returns *X*, and **w** is the vector of institution budget shares invested in each project, then project portfolio variance or risk is, by definition,

$$\begin{array}{c} Var\left[\text{ln}\left(U\right)\right]=\text{ln}\left(w\right){}^{\prime }\Sigma_{XX}\text{ln}\left(w\right) \end{array}$$ (22)

In the absence of data by which to calculate the project covariance matrix Σ_*XX*_, an AR4D institution may try to substitute the reverse engineered covariance matrix ${\tilde{\Sigma }}_{XX}$ (where it is assumed that project standard deviations have been estimated or crowdsourced, such that the reverse engineered project correlation matrix can be transformed into a covariance matrix), and then solve the problem

$$\begin{array}{c} \underset{w}{\text{min}}\;\;\;\text{ln}\left(w\right){}^{\prime }{\tilde{\Sigma }}_{XX}\text{ln}\left(w\right)\;\;\;s.t.\;\;\;1{}^{\prime }\text{ln}\left(w\right)=\text{ln}\left(U_{C}\right) \end{array}$$ (23)

Where *U*_*C*_ is the institution’s budget constraint, and **1** is a vector of ones. But note that the Lagrangian and first order conditions of this problem are then

$$\begin{array}{c} \begin{array}{cc} L & =\text{ln}\left(w\right){}^{\prime }{\tilde{\Sigma }}_{XX}\text{ln}\left(w\right)+\lambda \left(1{}^{\prime }\text{ln}\left(w\right)-\text{ln}\left(U_{C}\right)\right)\\ \nabla_{\text{ln}(w)}L & =2{\tilde{\Sigma }}_{XX}\text{ln}\left(w\right)+\lambda 1=0 \end{array} \end{array}$$ (24)

Where **0** is a vector of zeroes. Solving for the risk minimizing budget shares w* thus involves inverting the covariance matrix as follows.

$$\begin{array}{c} \text{ln}\left(w^{*}\right)=-\frac{\lambda }{2}{\tilde{\Sigma }}_{XX}^{-1}1 \end{array}$$ (25)

But the reverse engineered approximate covariance matrix ${\tilde{\Sigma }}_{XX}$ has *n* − *m* eigenvalues equal to zero (because it is calculated as the outer product of *m* retained loadings), and so is not invertible. The constrained risk minimization problem is thus ill posed.

On the other hand, the reverse engineered policy covariance matrix $\Sigma_{SS}^{↺}$ *is* invertible, thereby opening up the possibility of solving for the risk minimizing “policy weights”—i.e. the institution resource shares allocated to each policy. This may refer to actual funds, or may be interpreted more loosely as an allocation of attention, time, enthusiasm, political will, etc., as appropriate. Such weights are often assigned in a highly subjective, ad hoc manner. The method pursued thus far suggests the following, more rigorous approach. First, define the expected logged policy portfolio utility function as follows.

$$\begin{array}{c} E\left[\text{ln}\left(U\right)\right]=E\left[SB\right]{}^{\prime }\text{ln}\left(w\right) \end{array}$$ (26)

Where *E*[*SB*] is the vector of column means of the orthonormally rotated principal components *SB* (which in the AR4D context may be interpreted as the unobserved expected policy returns).

Then the policy portfolio risk follows as

$$\begin{array}{c} Var\left[\text{ln}\left(U\right)\right]=\text{ln}\left(w\right){}^{\prime }\Sigma_{SS}^{↺}\text{ln}\left(w\right) \end{array}$$ (27)

And the policy portfolio risk minimization problem can be formulated as

$$\begin{array}{c} \underset{w}{\text{min}}\;\;\;\text{ln}\left(w\right){}^{\prime }\Sigma_{SS}^{↺}\text{ln}\left(w\right)\;\;\;s.t.\;\;\;1{}^{\prime }\text{ln}\left(w\right)=\text{ln}\left(U_{C}\right) \end{array}$$ (28)

Where, in this case, the **w** are the policy weights. The Lagrangian and first order conditions are then

$$\begin{array}{c} \begin{array}{cc} L & =\text{ln}\left(w\right){}^{\prime }\Sigma_{SS}^{↺}\text{ln}\left(w\right)+\lambda \left(1{}^{\prime }\text{ln}\left(w\right)-\text{ln}\left(U_{C}\right)\right)\\ \nabla_{\text{ln}(w)}L & =2\Sigma_{SS}^{↺}\text{ln}\left(w\right)+\lambda 1=0 \end{array} \end{array}$$ (29)

With second order condition

$$\begin{array}{c} \begin{array}{c} \text{ln}\left(w\right){}^{\prime }\nabla_{\text{ln}(w)}^{2}L\text{ln}\left(w\right)>0\\ 2\text{ln}\left(w\right){}^{\prime }\Sigma_{SS}^{↺}\text{ln}\left(w\right)>0\\ 2Var[\text{ln}(U)]>0 \end{array} \end{array}$$ (30)

Which is always fulfilled. Note, moreover, that −λ reflects the institution’s budget shadow price, i.e., the marginal value of money (or attention, or enthusiasm, or whatever terms the budget is measured in) to the institution.

$$\begin{array}{c} \frac{\partial L}{\partial \text{ln}(U_{C})}=-\lambda \end{array}$$ (31)

And note that dotting the first order conditions through by ln(**w**) yields the following expression for λ.

$$\begin{array}{c} \begin{array}{cc} \text{ln}{\left(w\right)}^{\prime }\nabla_{\text{ln}(w)}L & =2Var\left[\text{ln}\left(U\right)\right]+\lambda \;\text{ln}\left(U_{C}\right)=0\\ \to \;\;\lambda & =-2\frac{Var[\text{ln}(U)]}{\text{ln}(U_{C})} \end{array} \end{array}$$ (32)

The institution’s budget shadow price is thus proportionate to the ratio of portfolio risk to cost.

$$\begin{array}{c} \frac{\partial L}{\partial \text{ln}(U_{C})}=2\frac{Var[\text{ln}(U)]}{\text{ln}(U_{C})} \end{array}$$ (33)

The first order conditions are then solved for the risk minimizing policy weights w* as follows.

$$\begin{array}{c} \begin{array}{cc} \text{ln}(w^{*}) & =\frac{\lambda }{2}\Sigma_{SS}^{↺-1}1\\ \text{ln}(w^{*}) & =-\frac{Var[\text{ln}(U)]}{\text{ln}(U_{C})}\Sigma_{SS}^{↺-1}1\\ w^{*} & =\text{exp}(-\frac{Var[\text{ln}(U)]}{\text{ln}(U_{C})}\Sigma_{SS}^{↺-1}1) \end{array} \end{array}$$ (34)

Where the ratio $\frac{Var[\text{ln}(U)]}{\text{ln}(U_{C})}$ is exogenously set by the institution in accordance with its risk tolerance.

The AR4D institution may also wish to experiment with a slightly different formulation of the resource allocation problem, replacing the policy covariance matrix with the policy correlation matrix (call this $K_{SS}^{↺}$). Because the policy variances are scaled to unity in the correlation matrix, the quantity $\text{ln}(\text{w})\prime K_{SS}^{↺}\;\text{ln}(\text{w})$ is less a reflection of portfolio risk than it is an indicator of portfolio net synergy, i.e., total synergy minus total tradeoffs, given a resource allocation **w**. Since net synergy is something desireable, the problem becomes a budget constrained synergy maximization problem, as opposed to a budget constrained risk minimization problem. Formally, this can be expressed as follows.

$$\begin{array}{c} \underset{w}{\text{max}}\;\;\;\text{ln}\left(w\right){}^{\prime }K_{SS}^{↺}\;\text{ln}\left(w\right)\;\;\;s.t.\;\;\;1{}^{\prime }\text{ln}\left(w\right)=\text{ln}\left(U_{C}\right) \end{array}$$ (35)

Finally, an AR4D institution might also be interested in applying these equations to the analogous problem of plant breeding, so as to find the risk minimizing (or synergy maximizing) allocation of program resources across a “portfolio” of plant traits.

## Conclusion

For a long time now, research institutions have faced increasing donor pressure to “do more with less” [7], “prove their relevance” [1], “show value for money” [8], and otherwise demonstrate “more efficient spending of resources” [9].

In response to this pressure, researchers have focused on the development of models for the ex-ante impact assessment of individual projects [5, 10–12]. However, new decision support tools are still urgently required at the portfolio level to determine optimal resource allocations across strategic objectives. In the absence of such tools, resource allocation procedures have been repeatedly undercut by stakeholder politics, institutional inertia, and other forms of subjective bias. The Consultative Group on International Agricultural Research, in particular, is said to “have a long history of good intentions but limited success in developing appropriate approaches for priority setting” [13]. And this, in turn, has contributed to unprecedented levels of toxicity in AR4D donor-researcher relations [13–15]. The toxicity is palpable across other disciplines as well [16, 17].

The task of allocating limited resources across strategic objectives that are all, in one way or another, vitally important, will never be an easy one. Nonetheless, it stands to reason that the introduction of objective, transparent, and inexpensive resource allocation mechanisms can substantially ameliorate the current atmosphere of distrust. As a step in this direction, above I have presented a novel project and policy synergy/tradeoff reverse engineering method based on principal components analysis. The proposed method aids in identifying areas in the AR4D portfolio where research impacts capitalize upon and enhance, or, conversely, annul and offset, each other.

The method can be applied to portfolios of projects or portfolios of policies. For policy portfolios, I showed how the reverse engineered policy covariance matrix may be used to solve for risk minimizing, or synergy maximizing, policy weights. These weights can then, in turn, inform the allocation of institution resources across research projects. I have also sketched out how the proposed method might be applied to analogous problems in plant breeding. The proposed method is not limited to these expository examples, nor even to the AR4D context, but rather applies to any portfolio level planning context where a relative lack of data is compensated by a relative abundance of domain expertise.

## Supporting information

S1 Data (CSV)

S1 Table Securities appearing in the financial example. (PDF)
