# Supplementary material for: “Reverse engineering” research portfolio synergies and tradeoffs from domain expertise in minimum data contexts
Source: PLoS One. 2021 Nov 12;16(11):e0259734. doi: 10.1371/journal.pone.0259734 (PMC8589176; doi:10.1371/journal.pone.0259734)
Supplement: S1 Table — (PDF) [file pone.0259734.s002.pdf]

S1 Table. Securities appearing in the financial example

Name	Symbol	Tracks
Financial Select Sector SPDR Fund	XLF	U.S. Financials Sector
Communication Services Select Sector SPDR Fund	XLC	U.S. Communications Sector
Consumer Discretionary Select Sector SPDR Fund	XLY	U.S. Luxury goods Sector
Consumer Staples Select Sector SPDR Fund	XLP	U.S. Consumer goods Sector
Health Care Select Sector SPDR Fund	XLV	U.S. Healthcare Sector
Technology Select Sector SPDR Fund	XLK	U.S. Technology Sector
SPDR Dow Jones REIT ETF	RWR	U.S. Real estate Sector
Utilities Select Sector SPDR Fund	XLU	U.S. Utilities Sector
Industrial Select Sector SPDR Fund	XLI	U.S. Industrial Sector
SPDR S&P Biotech ETF	XBI	U.S. Biotechnology Sector
iShares Transportation Average ETF	IYT	U.S. Transportation Sector
